# The Influence of Information Intervention Cognition on College Students’ Energy-Saving Behavior Intentions

**DOI:** 10.3390/ijerph17051659

**Published:** 2020-03-04

**Authors:** Ranran Yang, Chunxiao Yue, Jingjing Li, Junhong Zhu, Hongshu Chen, Jia Wei

**Affiliations:** 1 School of Management, Hefei University of Technology, Hefei 230009, China; yangranran@hfut.edu.cn (R.Y.); 2017214246@mail.hfut.edu.cn (C.Y.); jingjli@mail.hfut.edu.cn (J.L.);; 2 Research Center for Industrial Transfer and Innovation Development, Hefei University of Technology, Hefei 230009, China; 3 School of Economics and Finance, Xi’an Jiaotong University, Xi’an 710061, China

**Keywords:** energy-saving behavior intention, information intervention, perceived behavioral control, mediation effect, theory of planned behavior

## Abstract

Based on the theory of planned behavior, this research examines the influence of different types of information on the behavioral intentions of college students in the context of perceived behavioral control (perceived self-efficacy and perceptual control) as mediating variables. The results showed that: (1) Different types of information intervention factors have different effects on perceptual self-efficacy and perceptual control; the influence degree of economic cost has the strongest effect, followed by group pressure, while the influence degree of publicity and education has the weakest effect. However, policy intervention has no statistically significant effect on both of them (perceived self-efficacy and perceptual control). (2) Two variables, perceived self-efficacy and perceptual control, serve as mediators between information intervention factors and energy-saving behavior intention. (3) Individual characteristic factors have significant moderating effects on each path in the model of information intervention–perceived behavior control–intention. Finally, suggestions are made on how to encourage college students to more effectively save energy.

## 1. Introduction

As the world’s largest energy consumer and carbon emitter, China is facing severe energy and environmental pressure. The BP Energy Outlook 2019 edition pointed out that, although energy demand has slowed, China will still be the world’s largest energy consumer in 2040, accounting for 22% of global energy consumption. Oil import dependence will rise from 67% in 2017 to 76% in 2040. Dependence on gas imports will rise from 38% to 43% by 2040. In 2017, China emitted 9.23 billion tons of carbon dioxide, accounting for 27.6% of the global total [1]. To achieve sustainable and green development, the Chinese government has successively issued laws and regulations on Notice on Action Plan for Energy Saving, Emission Reduction and Low-carbon Development in 2014-2015, Notice on the Comprehensive Work Plan of Energy Saving and Emission Reduction during the 13th five-year Plan, Supplementary Notice on Comprehensive Demonstration of Energy Saving, and Emission Reduction Fiscal Policies, etc. The need to effectively encourage individuals to change consumption behaviors and adopt low-carbon practices is key to energy saving and emission reduction.

However, most of the existing research on individual-level energy-saving behavior and willingness focus on individual residents. For example, based on the theory of planned behavior, Ru et al. discussed the influence of normative factors and perceived behavior control on individuals’ willingness to save energy [2]. Zhang et al. used the structural equation model to analyze the influencing factors and mechanisms of urban residents’ habitual and purchasing energy-saving behavior [3]. Additionally, Van der Werf et al. tested the impact of personal commitment strategies (i.e., individuals’ commitment to change their behavior) on energy-saving behavior [4]. There is little research in this space on college students, a new force in energy saving and emission reduction. However, as a top energy-consuming group, college students’ consumption behavior has a degree of demonstration effect on their families, and the family is the basic unit of the community and the country [5]. Therefore, examination of college students’ energy-saving behavior is key in building a “two oriented society” (Two Oriented Society refers to resource saving society and environment-friendly society).

College students’ energy-saving is significantly different from that of non-student residents. On the one hand, school is the main activity location of college students, and the energy that students consume is provided by the school for free. Therefore, compared with non-student residents who pay for electricity and gas every month, the sensitivity of college students’ energy use is low, which to a certain extent propels energy waste. On the other hand, whether college students are in class or in life, they are in collective activities, and they are more vulnerable to the influence of group atmosphere than non-student residents. Further, college students generally have a herd mentality and strong plasticity [6]. At present, only a few scholars have explored the factors that affect college students’ environment, and there is a lack of sufficient demonstration on the functional relationship between different factors operating in this space [7,8,9]. In addition, the energy-saving behavior of college students has a degree of “knowing and doing separation,” that is, knowing is easy and doing is difficult. Most college students have a high energy-saving awareness or attitude, but the enforcement of energy-saving behavior is not high, there are some common implementation obstacles [6,8,9]. In view of this phenomenon, academics employ the concept of “perceptual behavior control” to reflect the difficulty degree of individual perceived behavior [10]. This article aims to solve the following three problems:

(1) What are the factors that make it difficult for college students to realize their energy-saving behavior potential?
(2) How can the difficulty of behavior perception of college students be reduced?
(3) Can perceived behavioral control improve college students’ willingness to save energy?

## 2. Literature Review and Model Hypothesis

Energy-saving behavior is generally divided into two types: habitual energy-saving behavior and purchasing energy-saving behavior [11,12]. Habitual energy-saving behavior generally refers to the reduction of some daily life behaviors and the change or adjustment of one’s habits, and these behavioral changes can help reduce energy consumption (e.g., turning off the lights after leaving, reducing air conditioning use, controlling air conditioning temperature, etc.). Purchasing energy-saving behavior refers to investing in new technology or energy-saving equipment to indirectly reduce energy use without changing lifestyle, such as purchasing energy-saving lamps [13]. Purchasing energy-saving behavior is influenced by the level of science and technology developments, product prices, subsidy policies, and other objective factors. Habitual energy-saving behavior can be controlled and changed through individuals’ subjective motivation [14]. Thus, there are different ways to study these two energy-saving behaviors [15]. In view of the limited energy purchase behaviors of college students, this article focuses on the habitual energy-saving behavior of college students.

The theory of planned behavior provides a new way to explain the general decision-making processes involved in individual behavior [10]. The theory points out that intention is the most direct factor to affect behavior, and the intention is the most predictive behavior tendency or motivation before behavior. The theory considers both external and internal factors that affect individual energy-saving behavior, including the attitude of individual internal behavior, and subjective norms and perceived behavior control. The theory has been widely used in research on green consumption behavior [16], green travel [17], employee energy-saving behavior [18], etc. Among these areas, the influence of perceptual behavior control on behavior intention and actual behavior has attracted increasing attention. For example, Karlijn et al. explored the factors driving young people’s energy behaviors based on the theory of planned behavior. The results from this work showed that perceived behavioral control directly affected the implementation of behavior [19]. Heath and Gifford, analyzing residents’ sustainable consumption behavior, found that perceptual behavior control was the most significant factor [20]. Shen et al. illustrated that besides personal moral obligation, perceptual behavior control was the most significant factor influencing intention to classify solid waste among 524 young people living in Hebei province, China [21]. Ru et al. investigated the behavioral intention to save energy among 450 eastern China residents and found that perceived behavioral control was the most important factor affecting behavioral intention to save energy [2]. However, at present, scholars mainly focus on measuring the relationship between perceived behavioral control and intention or behavior through questionnaire surveys, and there are few pre-influencing factors of perceived behavioral control, and hence, it is impossible to truly solve the problem of “high difficulty of perception.” Therefore, it is of great research value to determine the factors influencing of perceived behavior control of energy-saving behavior and intervene to change the perceived behavior control, and thereby, encourage changes in behavioral intention and energy-saving behavior.

In recent years, information intervention has become a focus in energy-saving behavior research. Many researchers have conducted information intervention research on individual energy-saving behavior [22,23,24]. The prevalence of information intervention strategies mainly comes from nudge theory of Thaler and Sunstein [25]. Information intervention is a specific application of nudge theory in the field of energy aimed at promoting energy-saving behavior. Based on data from 156 papers published between 1975 and 2012, Delmas et al. conducted a comprehensive analysis on the household energy-saving experiment based on information strategy, and found that average power consumption decreased by 7.4% [26]. Mi and Yang conducted a meta-analysis of 42 articles published between 1977 and 2014 regarding the influence of information intervention on energy-saving behavior and willingness, and concluded that information intervention strategy had a positive role in promoting energy-saving behavior and willingness in residents [27]. In terms of information intervention, sociology and cognitive psychology emphasize internal factors, and intervention behavior focuses on publicity, education, and persuasion. Furthermore, economics and application behavior emphasize external factors, and intervene behavior through policies, regulations, and taxes [28]. This article selects the external economic cost and policy intervention, internal publicity, and education and group pressure as the information intervention factors, and then analyzes the intervention path of various factors on college students’ energy-saving behavior intention.

Therefore, based on the theory of planned behavior, focusing on the factors of perceptual behavior control, this article establishes a theoretical model addressing the impact of information intervention on the habitual energy-saving behavior intention of college students, states research hypotheses, and uses questionnaire survey data to reveal the mechanism of different pre-intervention information on the habitual energy-saving behavior intention of college students.

### 2.1. Research Hypothesis on the Effect of Information Intervention Factors on Perceived Behavior Control

Perceptual behavior control refers to the degree of controllability perceived by an individual for a particular behavior; that is, the speculation and judgment on whether the individual has the ability to complete the behavior. At present, scholars divide perceived behavioral control into two independent and interrelated factors: self-efficacy of perceived behavioral difficulty and perceptual control of behavioral willingness [29].

#### 2.1.1. Research Hypothesis on the Effect of Information Intervention Factors on Self-Efficacy

Self-efficacy is the self-judgment of whether an actor can complete a certain behavior at a certain level. Different from behavioral control perception, self-efficacy reflects the control of the actor on external factors that affect their behavior. Self-efficacy reflects self-internalized cognition and judgment [30]. Bandura’s definition of self-efficacy is the degree of confidence people have in whether they can use the skills they have to complete a certain task; in turn, this depends on the external environment of the agent and similar behaviors they have practiced in the past. It is the perception of the agent’s ability to perform new behaviors [31]. Lu demonstrated that due to costs, it is more difficult to participate in energy-saving behaviors, and thus, residents’ willingness to do so is reduced. Conversely, when the economic cost is reduced, perceived self-efficacy of residents is weakened [32]. Shi found through empirical analysis that whether mandatory policy, incentive policy, or social policy, the implementation of the policy will improve residents’ low-carbon consumption ability, so as to increase the individual’s self-perception of implementing low-carbon behavior [33]. Yang et al. found that targeted publicity and education methods greatly enhance residents’ awareness of energy-saving behavior, thus, making it easier for residents to perceive the difficulty of participating in energy-saving behavior [34]. Chen et al. have illustrated that group pressure can lead to the opposite effect of self-attitude. Individuals will obtain group members’ approval and avoid punishment by maintaining behaviors consistent with those of the group [9]. Therefore, this article proposes the following hypothesis:

**Hypothesis 1** **(H1a):** Economic cost has a significant positive effect on perceived self-efficacy.

**Hypothesis 1** **(H1b):** Policy intervention has a significant positive effect on perceived self-efficacy.

**Hypothesis 1** **(H1c):** Propaganda education has a significant positive effect on perceived self-efficacy.

**Hypothesis 1** **(H1d):** Group pressure has a significant negative effect on perceived self-efficacy.

#### 2.1.2. Research Hypothesis of Information Intervention Factors on Perceptual Control

Perceptual control is an index that describes the degree of control of the agent over their own behavior. It judges whether the agent participates in the behavior from their own perspective, and their behavioral intention will not be easily changed by various factors [35]. In this article, perceptual control mainly refers to the control level in college students’ willingness to participate in habitual energy-saving behavior. From the perspective of individual behavior, there is a certain limit in self-control. When influenced by intervention information and perceptual control exceeds a certain critical point, the individual’s behavioral intention will change [35]. Regarding economic cost, research shows that the acceptance level of green consumption behavior premium is less than 5%, and when it exceeds 5%, green consumption intention will be significantly reduced [16]. In this article, the premium is less than 5%. At this time, as prices rise, residents will be affected by price sensitivity and other factors [32]; and to avoid spending more money, residents’ own perceptual control will become stronger. Mi et al. found that the formulation and implementation of relevant energy-saving policies and regulations ensures that residents have laws to follow when participating in energy-saving behaviors, and hence, can participate in energy-saving behaviors with more confidence and fulfilment [36]. Li et al. determined that when more targeted publicity of energy-saving behaviors was conducted, residents had a higher degree of recognition of energy-saving behaviors [35]. In a group-oriented culture, people are encouraged to obey the organization rather than pursue individual goals [37]. Therefore, this article proposes the following hypothesis:

**Hypothesis 2** **(H2a):** Economic cost has a significant positive impact on perceptual control.

**Hypothesis 2** **(H2b):** Policy intervention has a significant positive effect on perceptual control.

**Hypothesis 2** **(H2c):** Publicity and education had a significant positive impact on perceptual control.

**Hypothesis 2** **(H2d):** Group pressure has a significant negative effect on perceptual control.

### 2.2. Research Hypothesis of the Effect of Perceived Behavior Control on Behavioral Intention of Habitual Energy-Saving

Allen and Ferrand found a significant positive relationship between self-efficacy and pro-environment behavior [38]. Individuals with a stronger sense of self-efficacy are more inclined to set challenging goals, more likely to achieve a higher level of behavior, and have more endurance in the face of difficulties and obstacles. These individuals firmly believe in the realization of their own behavior and are willing to make an effort. On the contrary, individuals with low self-efficacy are more likely to quit when they encounter setbacks [30]. In other words, individuals tend to choose behaviors with high self-efficacy. Therefore, self-efficacy has a positive impact on behavioral intention for energy saving. On the other hand, literature suggests that improvement of perceptual control will enhance the control of behavioral intention; that is, individuals tend to choose the behavior with strong perceptual control [29,35]. Therefore, this article proposes the following hypothesis:

**Hypothesis 3** **(H3a):** Perceived self-efficacy has a significant positive impact on habitual energy-saving behavioral intention.

**Hypothesis 3** **(H3b):** Perceptual control has a significant positive effect on habitual energy-saving.

### 2.3. Hypothesis of Mediating Effect of Perceived Self-Efficacy and Perceptual Control

This article focuses mainly on the test whether perceived self-efficacy and perceived control have mediating effects between information intervention factors and habitual energy-saving behavioral intention. Therefore, this article puts forward the assumption:

**Hypothesis 4** **(H4a):** Perceived self-efficacy has a mediating effect between information intervention factors and habitual behavioral intention to save energy.

**Hypothesis 4** **(H4b):** Perceptual control has a mediating effect between information intervention factors and habitual energy-saving behavior intention.

### 2.4. Hypothesis of Moderating Effect of Personality Characteristic Variables

Many studies, most of which focus on gender, age, income, educational background, and occupational background, have assessed the influence of social demographic characteristics on energy-saving behavior. For example, Pothitou et al. [39] and Yu et al. [40] found that energy- saving behavior was significantly different based on gender factors, while Yang et al. argued that gender was not a significant factor influencing energy-saving behavior [41]. In terms of age, Belaid and Garcia [42] and Yang et al. [41] showed a non-linear relationship between age and energy-saving behavior. Income and education background have relatively inconsistent effects on energy-saving behaviors, which may be due to the inconsistent definition of energy-saving behaviors. For example, Yang et al.’s results showed that low-income groups are more willing to implement habitual energy-saving behaviors, while education background has no significant impact on myriad energy-saving behaviors [41]. Liu et al.’s research on Tianjin residents’ low carbon travel behavior showed that characteristics of various factors on the behavior have a direct influence. Further, on the basis of the analysis of the characteristic factors of the theoretical model of each path adjustment effect, the research shows that the demographic factors on the residents’ psychological factors into the process of behavior intention exists adjustment effect [43]. Based on this, combined with the characteristics of college students, this article proposes the following hypothesis:

**Hypothesis 5** **(H5a):** Gender has a significant moderating effect on various pathways in the information intervention–perceived behavior control–intention model.

**Hypothesis 5** **(H5b):** Grade level has a significant moderating effect on various pathways in the information intervention–perceptual behavior control–intention model.

**Hypothesis 5** **(H5c):** Major has a significant moderating effect on various pathways in the information intervention–perceived behavior control–intention model.

**Hypothesis 5** **(H5d):** Monthly allowance has a significant moderating effect on various pathways in the information intervention–perceived behavior control–intention model.

**Figure 1:**
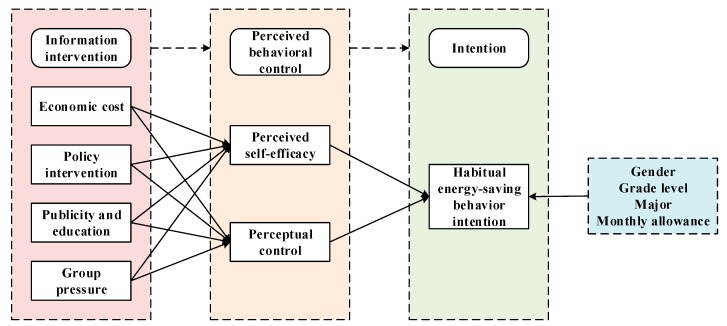
Research model on the influence of information intervention on college students’ behavioral intention to save energy.

In sum, the research model is established as shown in Figure 1, below. In this article, information intervention is regarded as an external stimulus that can stimulate individuals’ internal perception, while the internal perception of individuals, which is the individuals’ response to external stimuli (individuals’ sense of competence and autonomy), is instantiated in self-efficacy and perceptual control. In other words, information intervention (external stimuli) could strengthen individuals’ perceived self-efficacy and perceptual control (emotional and psychological reactions) that generate intention of habitual energy-saving behavior (motivation and behavioral reactions).

## 3. Materials and Methods

### 3.1. Questionnaire Design

The design of the questionnaire items is on the basis of the above model, some references for domestic and foreign relevant maturity scale [2,5,15,16,30,32,33,44,45,46], and combined with the specific situation of the college students. The questionnaire is mainly divided into four parts: basic information description, behavioral intention to save energy, perceived behavioral control, and information intervention. In the process of questionnaire design, relevant experts were invited to discuss the problem setting, and the questionnaire was modified and improved according to expert opinions. At the same time, to ensure high questionnaire reliability and validity, we conducted a pre-survey to test the initial questionnaire. According to the data analysis results, we modified and deleted the index items with errors to form a formal questionnaire. The specific item design is shown in Appendix A. Moreover, a 5-point Likert scale was used in our questionnaire to measure the scores of items, with 1 = “strongly disagree”, 2 = “disagree”, 3 = “neutral”, 4 = “agree”, and 5 = “strongly agree”.

### 3.2. Distribution and Recovery of Questionnaires

This article adopted the method of an online questionnaire and offline field distribution to formally distribution the questionnaire. The research subjects were college students in Hefei city, Anhui province. Hefei is the important center for technology and education in China and the innovation capital with great international influence. According to the statistics of the Chinese bureau of education, by the end of 2018, there were 55 colleges and universities and more than 600,000 college students in Hefei, and it ranked 11th among Chinese cities. On the other hand, urban per capita disposable income and per capita consumption expenditure of Hefei is nearly the national average. According to the statistics of National Bureau of Statistics of China, in 2018, national urban per capita disposable income and per capita consumption expenditure are 39,251 CNY and 26,112 CNY, and the data of Hefei are 41,484 CNY and 25,339 CNY respectively. Therefore, Hefei has some representation as the research subject. The formal investigation lasted for one week. In total, 230 questionnaires were distributed and all were recovered; 221 questionnaires were effective with an effective rate of 96.1%, and the number of effective questionnaires was larger than the minimum sample size of 159 (the error range was 5%). Analysis of the characteristics of the recovered effective questionnaires (Table 1) indicated that the ratio of male and female students was comparable, and the proportion of students in different grade levels was not significantly different, which minimizes the influence of gender and grade level factors on the data, and ensures the objectivity of the questionnaire. Science and engineering account for 83.26% of the academic majors, which conforms to the distribution ratio of academic majors of Hefei University students. In terms of monthly living expenses, 84.17% of the surveyed college students spent less than 1600 CNY per month. Referring to the minimum wage standard of 1550 CNY per month in Hefei in 2018, this suggests that college students may be a low-consumption group.

## 4. Results and Discussion

We used the structural equation model (SEM) to examine the research model. This SEM analysis is a robust and influential high quality multivariate statistical analysis technique to examine the model with latent variables, and for parameter assessment and hypothesis testing e.g., factor analysis and regression or path analysis [47,48]. In this article, SEM is performed by using AMOS 24.0 which is a powerful and widely used software produced by IBM (Armonk, NY, USA) that explores mutual relations among different variables. We firstly tested the reliability and validity of the measurement model, and then examined the structural model to test research hypotheses.

### 4.1. Reliability and Validity Analysis

Scale reliability, which referred to the consistency of the indicators, was measured using Cronbach’s alpha coefficient and combined reliability [47], and the reliability test results are shown in Table 2. As can be seen from Table 2, the Cronbach’s alpha value of each variable is in the interval of 0.707 to 0.916, and the CR value ranges from 0.874 to 0.941, far higher than the theoretical minimum value of 0.7, indicating that the overall measurement results of the questionnaire had a good reliability [49].

In terms of questionnaire validity, the questionnaire in this article mainly refers to relevant mature scales and was consulted on by experts, which can help ensure high validity. Meanwhile, construct validity, convergence validity, and discriminant validity were detailed analyzed as follows.

Construct validity was tested by measuring factor loading and cross loading of each item using AMOS 24.0. As shown in Table 2, the factor loading values of all measurement items are higher than 0.50, and the measurement results show that the factor loading is larger than the cross loading. Therefore, the questionnaire had a high construct validity [49].

Convergence validity was measured using factor loading, combined reliability (CR), and average variance extracted (AVE). As shown in Table 2, the factor loading value of the items ranges between 0.765 and 0.903, above the minimum threshold 0.7. The measured values of CR are in the interval of 0.874 and 0.941, above the minimum threshold 0.7. The values of AVE range from 0.656 to 0.800, above the critical value of 0.5, which indicates that the questionnaire had a high convergence validity [49].

Discriminant validity was tested by the square root of AVE. According to the Fornell–Larcker criterion, the square root of AVE should be greater than the correlations between the variables [49]. As the calculation results shown in Table 3, the bold text on the diagonal in the table represents the square root of AVE, and the non-diagonal lines are the correlation coefficients between variables. This indicates that the questionnaire had a high discriminant validity.

### 4.2. Structural Equation Model Test

After the establishment and identification of the structural equation model, AMOS 24.0 was used to estimate and test the initial structural equation model. As indicated in Table 4, the final structural model reached a good fit, with all the indices conforming to the reference values in Bagozzi and Yi [50].

The results of the modified model path/load analysis are shown in Table 5. All the path coefficients are statistically significant except that of policy intervention to perceived self-efficacy (β = −0.084, CR = −0.851, *P* > 0.05) and policy intervention to perceptual control (β = −0.008, CR = −0.076, *P* > 0.05). Moreover, it can be seen that different types of information intervention factors have different effects on perceived self-efficacy and perceptual control. Specifically, among the supported hypotheses, economic cost is the most important factor affecting perceived self-efficacy (β = 1.734, CR = 4.426, *P* < 0.001) and perceptual control (β = 1.688, CR = 4.484, *P* < 0.001). This is also consistent with the characteristics of college students. According to the characteristics of 84.17% of the sample, college students’ monthly living expenses under 1600 CNY, the consumption level of college students is generally low. Because there is no source of economic income, students’ consumption mainly depends on family support, and thus, they are more sensitive to the economic cost of energy saving and emission reduction. Propaganda and education are the least influential factors on perceived self-efficacy (β = 0.550, CR = 4.904, *P* < 0.001) and perceptual control (β = 0.406, CR = 3.322, *P* < 0.001). On the one hand, it takes considerable time to change the concept of individual energy saving and emission reduction with the help of publicity and popularization, making it difficult to achieve significant results in the short-term. On the other hand, the cost-benefit correlation between publicity and education, and energy saving and emission reduction of college students is not directly affected by economic factors, and the incentive effect is weak. Regarding the significant negative influence of group pressure on perceived self-efficacy (β = −1.036, CR = −2.778, *P* < 0.01) and perceptual control (β = −1.108, CR = −3.023, *P* < 0.01), it is also consistent with the characteristics of college students. Compared with individual non-student residents, college students are almost always in a collective life, and they are more likely to be influenced by the group, which may affect their judgment.

The conclusion that policy intervention has no significant effect on perceived self-efficacy and perceptual control is different from research conducted with urban residents. For example, Shi shows that different policy interventions have a significant impact on residents’ energy-saving behavior [33]. Yue Ting found that the implementation of policies can strengthen residents’ willingness to conduct energy saving [30], etc., but these studies are all targeted at urban residents. Although college students’ policy interventions on energy-saving behavior and willingness may be known through the Internet, teachers, classmates and other channels, students lack practical experience, and their policy acceptance is very limited. Therefore, it is difficult for them to generate a personal sense of identity. For example, Zhang and Zhang found that college students are in a relatively fixed living environment, with a relatively weak sense of social responsibility, and they will be a mind of indifference [51]. To a certain extent, this also explains why the effect of policy intervention is not significant for college students.

### 4.3. Analysis of Mediating Effects

According to Baron and Kenny [52], the mediating effect test can be divided into the following four steps: (1) discuss the significance (*c*) value of independent variable and dependent variable; (2) discuss the significance (*a*) value of independent variable and intermediate variable; (3) discuss the significance (*b*) value between the mediating variable and the dependent variable. At this point, the significant values of (*c*), (*a*), and (*b*) indicate that the mediating variables play an intermediary role, and then further discussion plays a role of complete mediation or partial intermediary role; (4) discuss the establishment of regression model by using independent variables and intermediate variables to jointly predict the significance of (*c’*) value of the significance of dependent variables. The significant (*c’*) correlation indicates that the model plays a partial mediating role, otherwise it is a complete mediating role.

The mediating effect was analyzed by bootstrap (self-sampling 2000 times) results in AMOS 24.0. After the model operation, the data is iterated to the 14th time to get convergence, at this time the *c*, *a*, and *b* values of significance are shown in Table 6. Policy intervention factors have no significant influence on perceived self-efficacy and perception control, so the mediation inspection will no longer discuss the information interference factors. The analysis shows that *a* values of group pressure, publicity and education, and economic cost are significant (*p* < 0.01). Additionally, the *b* values of perceived self-efficacy and perceptual control are also significant (*p* < 0.01). This result indicates that the model has a statistically significant mediating effect. Further check the value of (*c’*), as shown in Table 6, the value of (*c’*) is also significant (*p* < 0.01). Therefore, we may know that perceived self-efficacy and perceptual control play a partial mediating role in the paths from economic cost, publicity and education, group pressure to intention of exhibit habitual energy-saving behavior.

### 4.4. Analysis of the Influence of Individual Characteristics on Energy-Saving Behavioral Intentions and Paths

The influence of individual characteristics of gender, grade level, academic major, and monthly living expenses on behavioral intention to save energy was tested using variance analysis (One-way ANOVA). Prior to ANOVA modeling, homogeneity of variance testing was required to determine whether ANOVA was appropriate (IBM SPSS 22.0, Armonk, NY, USA). The results are shown in Table 7. None of the variables were statistically significant (*p* > 0.05), and the variance was homogeneous; thus, suitable for variance analysis.

Gender, grade level, major and monthly allowance were used as independent factors and energy-saving behavior intention was the dependent variable. One-way ANOVA was conducted using SPSS 22.0, and the results showed that only different grade levels had a significant difference (P*grade* = 0.040 < 0.05) in energy-saving behavior intention. Gender, major and monthly allowance had a significant influence (P*gender* = 0.254, P*major* = 0.835, P*ma* = 0.319) on behavioral intention to save energy. This conclusion is consistent with the research conclusions of Yang et al. [36] and Belaid and Garcia [37]. Specifically, the mean difference of energy-saving behavior intention among different grade levels was ranked from the highest to the lowest in the following order: freshman (4.33) > senior (3.73) > junior (3.56) > sophomore (3.44). Freshmen had the highest energy-saving behavior intention, sophomores had the lowest, and there were significant differences among grade levels.

In addition, on the basis of analyzing the direct influence of personality characteristics on intention, this article further analyzed the influence degree of personality characteristics on the paths in the model; that is, the moderating effect of demography was analyzed by the multi-path grouping method. The specific results are shown in Table 8. To facilitate the statistical analysis, this article defines the students in the first and second year of university as junior grade, and the students in the third and fourth year of university as senior grade. The majors are divided into three groups: liberal arts, science, and engineering. Monthly allowance higher than 1200 are divided into high consumption. Monthly allowance below 1200 CNY are divided into low consumption.

As shown in Table 8, individual characteristic variables on each path have a specific regulation effect. But because of the more significant differences between the groups, compared with the perceived self-efficacy, individual characteristic variables adjustment mainly embodies in containing perceptual control path. Specifically, male students, junior grade, liberal arts students, and low consumption students are more likely to significantly affect their perceptual control because of the changes of their economic factors, the degree of contact of publicity and education, the degree of perception of group pressure, and other information intervention factors. For example, the difference between male (β = 0.54, *P* < 0.01) and female (β = 0.03, *P* > 0.1) on EC→PC was very significant. Moreover, it is also easier to significantly improve their habitual energy-saving behavior intention because of the perceptual control improvement. Therefore, this group should be the main target of information interventions, because this group has better cognitive and behavioral transformation effects of information intervention, and the stimulation effect will be better after implementation.

## 5. Conclusions and Recommendations

### 5.1. Research Conclusions

The main conclusions are as follows: (1) different types of information intervention factors have different effects on the perceived self-efficacy and perceptual control. Economic cost, group pressure, and propaganda education have significant effects on perceived self-efficacy and perceptual control. Among them, the degree of influence of economic cost is the strongest, followed by group pressure, and the degree of influence of publicity and education is the weakest. However, policy intervention factors have no significant effect on perceived self-efficacy and perceptual control. (2) Perceived self-efficacy and perceptual control have significant positive effects on habitual energy-saving behavior intention. (3) Perceived self-efficacy and perceived control have a partial mediating effect between information intervention factors and habitual energy-saving behavioral intention. Additionally, information intervention factors have a direct effect on habitual energy-saving behavior intention. (4) Among the individual characteristic factors such as gender, grade, major and monthly living expenses, only the grade factor has a direct significant correlation with the habitual energy-saving behavior intention of college students (non-linear relationship). However, almost all individual characteristic factors have significant moderating effects on each path in the model of information intervention–perceived behavior control–intention.

### 5.2. Policy Suggestions

Based on the above conclusions and the characteristics of college students, this article proposes the following strategies and suggestions to improve the willingness of college students to save energy:

(1) Economic incentive activities. Since economic cost has a great impact on the college students in the low-consumption group, the school or class can give material rewards to energy-saving behavior markers, such as distributing small gifts, to stimulate improvement of college students’ energy-saving behavior intention in the economy or in-kind.
(2) Establish a notification system of energy-saving behavior. The establishment of a timely information notification system, weekly or monthly timely notification; especially dormitory electricity saving and the corresponding energy and environment improvement, to enable college students to better understand energy-saving behavior and its significance, to enhance their willingness to engage in energy-saving behaviors. On the other hand, group pressure will significantly affect college students’ behavioral intention to save energy. Therefore, this potential advantage should be given full play to shape the energy-saving atmosphere in schools and improve individual behavioral intention to save energy under group pressure and supervision.
(3) Carry-out targeted publicity and education activities. Although there are many publicity activities, such as having an energy-saving month and energy-saving slogans on campus, participation and awareness of college students are not high enough in practical applications. Many of these efforts fail to capture the attention and interest of college students. Therefore, more colorful and interesting publicity methods of energy-saving are needed, such as one-hour energy-saving activities on earth or campus exchange activities organized out of dormitory that night.
(4) Enhance the sense of experience and identity in the implementation of policies and systems. Due to the limitation of campus activities, college students lack practical experience and experience in energy-saving policies, and their degree of acceptance is very limited, which makes it difficult to have a personal sense of identity. Therefore, the school can enrich students’ social practice activities and extracurricular cognition, improve their sense of social responsibility, make students feel and understand policies, and enhance their sensitivity to policy intervention.
(5) The implementation of information intervention should focus on males, junior grade, liberal arts students, and low consumption college students, because they are more likely to affect their perceptual behavior control due to the received information intervention, and then affect their willingness to adopt energy-saving behavior. Besides, one group leads another group to gradually improve the information acceptance and behavior transformation power of female, senior, science and engineering, and high-consumption university groups.

### 5.3. Deficiency and Prospect

Limited by data availability, this study still has the following shortcomings: First, the establishment of the model only examines the willingness of energy-saving behavior; the path from the willingness to energy-saving behavior is not considered. There may be changes in intervention information in this process, which makes the final energy-saving behavior produce different differences. Second, the results cannot be well generalized to the entire population as our sample is composed by university students. Third, we did not show to respondent different scenarios as in a stated preference survey. Future research must address these limitations.

## Figures and Tables

**Table 1:** Basic respondent characteristics.

Options		Sample Size (Proportion)	Options		Sample Size (Proportion)
Gender	male	118 (53.4%)	Grade level	Freshman year	38 (17.2%)
	female	103 (46.6%)		Sophomore year	48 (21.7%)
Monthly allowance (yuan)	<800	13 (5.9%)		Junior year	60 (27.2%)
	800–1200	85 (38.5%)		Senior year	75 (33.9%)
	1200–1600	72 (32.6%)	Major	The liberal arts	33 (14.9%)
	1600–2000	29 (13.1%)		science	103 (46.6%)
	>2000	22 (9.9%)		engineering	81 (36.7%)
				medical	4 (1.8%)

**Table 2:** Reliability and validity.

Latent Variable	Measurement Item	Loading	Cronbach’s Alpha	Combined Reliability	Average Variance Extracted
Habitual energy-saving behavior intention (HEBI)	HEBI1	0.800	0.865	0.905	0.656
	HEBI 2	0.781			
	HEBI 3	0.765			
	HEBI 4	0.809			
	HEBI 5	0.890			
Perceived self-efficacy (PSE)	PSE 1	0.840	0.860	0.905	0.705
	PSE 2	0.835			
	PSE 3	0.840			
	PSE 4	0.843			
Perceptual control (PC)	PC 1	0.853	0.826	0.898	0.745
	PC 2	0.878			
	PC 3	0.859			
Economic cost (EC)	EC 1	0.882	0.714	0.875	0.778
	EC 2	0.882			
Policy intervention (PI)	PI 1	0.872	0.884	0.921	0.745
	PI 2	0.872			
	PI 3	0.842			
	PI 4	0.866			
Publicity and education (PE)	PE 1	0.903	0.916	0.941	0.800
	PE 2	0.902			
	PE 3	0.891			
	PE 4	0.882			
Group pressure (GP)	GP 1	0.881	0.707	0.874	0.776
	GP 2	0.881			

**Table 3:** Results of discriminant validity.

Latent Variable	HEBI	PSE	PC	EC	PI	PE	GP
HEBI	**0.810**						
PSE	0.560 ***	**0.840**					
PC	0.418 ***	0.423 ***	**0.863**				
EC	0.449 ***	0.423 ***	0.339 ***	**0.882**			
PI	0.401 ***	0.380 ***	0.309 ***	0.403 ***	**0.863**		
PE	0.471 ***	0.417 ***	0.341 ***	0.415 ***	0.453 ***	**0.894**	
GP	0.371 ***	0.356 ***	0.262 ***	0.368 ***	0.428 ***	0.384 ***	**0.881**

Note: *** *P* < 0.01.

**Table 4:** Fitting index of the modified structural equation model.

Fitting Index	Fitting Index Value	Best Standard	Fitting Evaluation
Chi-Square (χ²)	551.071	the smaller the better	-
degrees of freedom (df)	235	The bigger the better	-
Chi-Square/df	2.345	1 < NC < 3	ideal
Parsimonious Normed Fit Index (PNFI)	0.719	>0.50	ideal
Comparative Fit Index (CFI)	0.903	>0.90	ideal
Incremental Fit Index (IFI)	0.904	>0.90	ideal
Root Mean Square Error of Approximation (RMSEA)	0.074	<0.05	Relatively ideal

**Table 5:** Revised model path/load analysis results.

Regression Path	Estimate	S.E.	C.R.	P	Correspondence Hypothesis	Verification Results
EC→PSE	1.734	0.392	4.426	0.000	H1a	Verified
EC→PC	1.688	0.377	4.484	0.000	H2a	Verified
PI→PSE	−0.084	0.099	−0.851	0.395	H1b	Failed
PI→PC	−0.008	0.112	−0.076	0.940	H2b	Failed
PE→PSE	0.550	0.112	4.904	0.000	H1c	Verified
PE→PC	0.406	0.122	3.322	0.000	H2c	Verified
GP→PSE	−1.036	0.373	−2.778	0.005	H1d	Verified
GP→PC	−1.108	0.367	−3.023	0.003	H2d	Verified
PSE→HEBI	1.226	0.257	4.765	0.000	H3a	Verified
PC→HEBI	0.506	0.245	2.064	0.039	H3b	Verified

**Table 6:** Double-tailed test results based on BC (Bias-Corrected) method.

The Path	The Total Effect (*c* value)	Direct Effect (*a*, *b* value)	The Indirect Effect (*c’* value)
EC→PC	0.000	0.000	---
EC→PSE	0.000	0.000	---
EC→HEBI	0.001	---	0.001
GP→PC	0.000	0.000	---
GP→PSE	0.000	0.000	---
GP→HEBI	0.002	---	0.002
PE→PC	0.002	0.002	---
PE→PSE	0.001	0.001	---
PE→HEBI	0.001	---	0.001
PC→HEBI	0.027	0.027	---
PSE→HEBI	0.001	0.001	---

**Table 7:** Tests of homogeneity of variance.

Individual Characteristics	Levene Statistic	df1	df2	Significant
Gender	1.939	1	190	0.165
Grade level	3.366	3	188	0.065
Major	1.080	3	188	0.359
Monthly allowance	2.309	4	187	0.060

**Table 8:** Results of multi-group analysis.

Path	Gender		Grade Level		Major			Monthly Allowance	
	Male	Female	Junior	Senior	Arts	Science	Engineering	Lower	Higher
EC→PSE	1.54 ***	1.49 ***	0.87 ***	1.92 ***	1.26 ***	1.98 ***	1.40 ***	1.72 ***	1.05 ***
EC→PC	0.54 ***	0.03	0.53 **	0.19	0.89 **	0.25	0.2	0.44 **	0.16
PE→PSE	0.87 ***	0.62 ***	0.82 ***	0.76 ***	0.81 ***	0.72 ***	0.82 ***	0.87 ***	0.61 ***
PE→PC	0.29 ***	0.01	0.49 **	0.06	0.44 ***	0.09	0.11	0.21 **	0.07
GP→PSE	0.76 ***	0.70 ***	0.73 ***	0.73 ***	0.77 ***	0.61 ***	0.83 ***	0.97 ***	0.42 ***
GP→PC	0.22 ***	0.01	0.43 *	0.04	0.26 *	0.05	0.09	0.22 *	0.03
PSE→HEBI	0.87 ***	0.85 ***	0.92 ***	0.75 ***	0.78 ***	0.85 ***	0.77 ***	0.87 ***	0.67 ***
PC→HEBI	0.84 ***	0.61 ***	0.92 ***	0.62 ***	0.78 ***	0.59 ***	0.79 ***	0.85 ***	0.47 ***

Note: *** *P* < 0.01, ** *P* < 0.05, * *P* < 0.1.

## Appendix A

**Table A1:** Research instrument.

Latent Variables	Questions
Daily energy-saving behavior intention	I think I have a high desire to save energy.
	When I finally leave the classroom or dormitory, I will pay attention to turning off the lights.
	When using the air conditioner, I am willing to adjust to the optimum temperature of 26 ℃, not too high or too low.
	I am willing to use air conditioning in moderation, and try to increase or decrease clothing to adapt to room temperature.
	When travel conditions allow, I would like to choose more public transportation, bicycle or walking modes.
Perceived self-efficacy	In life, I find it easy to implement energy-saving behavior, so there is a high willingness to save energy.
	When I plan to conduct energy-saving behaviors, I can clearly recognize the difficulty I perceive in this behavior.
	Although the actual number of times I participated in energy-saving activities is not many, I always maintain the enthusiasm and confidence to participate in energy-saving activities.
	I think I have a higher willingness to save energy than my classmates around me.
Perceptual control	I think I can stick to an energy-saving behavior for more than three months.
	I am more supportive of energy-saving behavior. Although it is restricted by various factors (such as time, economy, etc.), I can do my best.
	My desire to save energy will not be easily changed by external influences.
The economic costs	My first concern is cost performance, but also environmental protection.
	If the cost premium for participating in energy-saving activities is within my affordable range (generally 5%), I still have a strong desire to save energy.
Policy intervention	The standardization and perfection of the rules and regulations of the school on energy-saving behavior will make me more willing to save energy.
	The school’s mandatory regulations on energy-saving behavior will make me more willing to save energy.
	If I set rewards (commendations or prizes) for energy-saving behaviors, I will be more willing to save energy.
	The timely disclosure of energy-saving information in the dormitory (once a week) will make me more willing to save energy in the dormitory.
Publicity and education	Good media publicity activities on energy-saving behavior will inspire me to have a higher desire to save energy.
	The publicity of energy-saving behavior by the school and the media can help me better understand the connotation of energy-saving behavior and thus inspire me to be more willing to save energy.
	Public publicity can make me pay more attention to environmental protection and energy saving, and inspire me to have a higher desire to save energy.
	The longer and more comprehensive the publicity of energy-saving behavior, the more attention I will pay to environmental protection, so as to inspire me to be more willing to save energy.
Group pressure	I often change my intention to save energy and give up energy-saving behavior for reasons such as saving face, even though my intention to save energy is very high at this time.
	When my friends and relatives do not participate in the energy-saving behavior, I will give up the energy-saving behavior even though I have the intention to save energy.
